# Detailed Analysis of Immune Tolerance Mechanisms to SARS-CoV-2 in Children Is Needed

**DOI:** 10.3389/fped.2021.652838

**Published:** 2021-11-02

**Authors:** Hiroshi Ehara

**Affiliations:** Pediatrics and Primary Care, Ehara Clinic, Tokyo, Japan

**Keywords:** coronavirus, immunotolerance, pediatrics, cytokine storm, COVID-19

## Introduction

In December 2019, a novel coronavirus, severe acute respiratory syndrome coronavirus 2 (SARS-CoV-2) emerged in Wuhan, Hubei Province, China, and caused the coronavirus disease 2019 (COVID-19) pandemic. As of December 17, 2020, over 74 million cases have been reported worldwide, contributing to more than 1.6 million deaths (1). COVID-19 presentation ranges from mild flu-like symptoms to fatal respiratory failure, shock, and multiorgan dysfunction. Surprisingly, children exhibit milder symptoms than adults, and most cases are asymptomatic (2). This is consistent with the severe acute respiratory syndrome coronavirus (SARS-CoV) and Middle East respiratory syndrome coronavirus (MERS-CoV) outbreaks, which had high mortality in adults but none in pediatric patients (3, 4). As the immune system changes drastically with aging, we hypothesize that determining the immune response to COVID-19 in children is key to understanding disease susceptibility, determinants of disease severity, and therapeutic candidates for COVID-19. However, data on factors that protect children from severe COVID-19, particularly immune tolerance mechanisms in children infected with SARS-CoV-2, are limited.

## Viral Clearance

Some studies suggest that children mount a more effective immune response to SARS-CoV-2, resulting in enhanced viral clearance. Pediatric patients have higher serum concentrations of IL-17A and IFNγ than adult patients shortly after COVID-19 presentation, which may contribute to early immune protection (5). Aging impairs the expression of pattern recognition receptors on monocytes (6) and the number and function of invariant NKT cells (7), which could reduce IFNγ and IL-17 levels, respectively. Together, the findings of these studies are consistent with the hypothesis that more robust innate immune response in children helps in early clearance of SARS-CoV-2, which can further reduce adaptive immune response. In support of this hypothesis, some studies show that the adaptive immune response to SARS-CoV-2 in children is reduced, as shown by lower frequencies of spike-specific CD4 T cells, neutralizing antibody titers, and antibody-dependent cellular phagocytosis (5, 8). Also, children lack nucleocapsid-specific antibodies that require lysis of virally infected cells (8), supporting enhanced clearance early in infection.

Other studies have demonstrated that children exposed to SARS-CoV-2 have T cell and antibody responses similar to that in adults (9, 10). However, the majority of children remained PCR negative and developed minimal or no symptoms, suggesting a more effective adaptive immune response and viral clearance. Children have a higher proportion and number of T and B cells, and naïve T cells reduce in quantity with aging (11). Adults infected with SARS-CoV-2 have reduced lymphocyte counts (12); therefore, the higher numbers of lymphocytes, particularly naïve T cells, in children could allow for a stronger T cell-mediated response against SARS-CoV-2. Another hypothesis is that children have more cross-reactive T and B cell immunity, possibly as a result of frequent and recent infections by related coronavirus strains or recent vaccination for diphtheria, tetanus, and whooping cough (13, 14). The importance of pre-existing common-coronavirus immunity in controlling SARS-CoV-2 infection became apparent when humoral memory against common β-coronavirus OC43, a virus circulating every year, was found to be associated with the acutely evolving SARS-CoV-2 disease scenario. The SARS-CoV-2 S2-specific humoral immune response in survivors occurred in parallel with the expansion of existing class-switched OC43-specific humoral immunity from IgM to IgG. This humoral immunity selectively expanded in asymptomatic SARS-CoV-2 infected individuals, leading to a more robust containment of the infection. Especially, considering conservation of S2 across β-coronaviruses, presence of S2-specific antibodies with Fc-receptor binding capabilities strongly predicted the protective immunity against mortality (15).

However, preliminary studies suggest that these cross-reactive antibodies do not bind to the SARS-CoV-2 receptor binding domain and are therefore not neutralizing (16). Furthermore, cross-reactive T cells are more abundant in the elderly and may be a risk factor for severe COVID-19 (17).

## Immunotolerance

Some studies suggest that children have viral loads similar to adults (18, 19), suggesting that milder symptoms in children may not be a result of enhanced viral clearance. In most cases of COVID-19, the immune response can effectively clear the virus. However, a dysfunctional immune response can manifest in patients with severe COVID-19 and fatal respiratory failure, resulting in excessive systemic inflammation and cytokine storm, which can cause severe lung and multiorgan damage (20). Patients with COVID-19 exhibit elevated cytokine levels, specifically IL-6 and TNF-α, which are higher in those admitted to the ICU (21). Also, IL-6 and TNF-α serum levels are predictors of disease severity and death (22). Therefore, COVID-19 disease severity is not only associated with the virus itself but also the host response. Interestingly, hospitalized children had lower concentrations of IL-6 and TNF-α than adults with more severe outcomes (5). Immunotolerance mechanisms in children may not influence viral burden but may reduce the damage caused by a dysfunctional immune system.

The neonatal immune system is marked by its flexibility and hypervigilance as well as heightened responsiveness to numerous stimulants. However, the varying developmental stages train the infantile immune system to be highly tolerant to stimulatory antigens. During fetal life development, the immune system becomes functionally programmed to promote tolerance to the maternal environment *in utero*. The rapidly changing environmental and microbial exposures during the postnatal period relentlessly stimulate immune development, requiring tightly regulating perinatal inflammatory reactions (23). The tightly regulated mechanism also helps to develop selective tolerance toward specific commensal microbial components by suppression of rejection while discerning the need of eliminating other components through vigorous host responses during the neonatal and infantile period (24). Thus, an intricately regulated perinatal inflammatory reaction facilitating optimal host immune develops *via* crosstalk between microbes and their hosts. Neonatal tolerogenic immune responses are controlled partially by adaptive regulatory T cells (Tregs) as well as pronounced pattern-recognition receptors (PRR)-mediated IL-10 production by neonatal APCs (25). Satisfying such complex demands of early life is achieved in part through the development of multiple highly effective immune regulatory strategies in parallel with immune effector functions in early life (25). The regulatory innate lymphoid cells (ILCregs) in the mucosal and cutaneous barriers are considered to play an important role in protecting neonates. ILCregs also produce IL-10 and TGF-b1 during intestinal inflammation to contribute to the resolution of innate intestinal inflammation (26). CD71+ cells, which are absent in most healthy adults, express multiple factors, including arginase-2, which are essential for immunosuppression in neonates (27). CD71+ cells also promote the development and function of Tregs, which are more numerous in infants and children (28). A higher frequency of myeloid-derived suppressor cells (MDSC) is also found in neonates (29).

The potential benefit of such attenuated immune responses in early life is evident for hepatitis B virus infection, where the immune regulatory mechanisms are dominant during early life to prevent the immune-mediated harm observed in infected adults. The lower mortality rate of children than adults from Influenza virus infection could also be explained by lessened inflammatory responses in the lung (30). HIV-infected pediatric slow progressors (PSPs) or the children showing suppressed immune response and slower disease progression while harboring on-going viral replication by preserving CD4 T-cell occurs through suppression of immune activation. Suppressive memory Treg proliferation and IL-10 secretion are increased in PSPs, requiring both immune regulations *via* Treg activity and IL-10 production, and enhancement of T-cell homeostatic IL-7 signaling (31).

The process of change in immune response from the innate to the adaptive immunity type might include errors in regulatory mechanisms. Concurrent accumulation of senescent or exhausted cells is associated with enhanced proinflammatory cytokines production and the recruitment of immune cells, resulting in an imbalance toward inflammaging of the human immune system (32).

Dysregulation of immune homeostasis is preceded by the proinflammatory state of aged T cells. Patients with AIDS showed an accelerated T cell senescence. Tregs senesce preferentially over conventional T cells (Tconv cells) through DCAF1/GSTP1/ROS–dependent mechanisms. This results in an increasing imbalance between the function of Tregs and Tconv cells during aging. Considering Tconv cells activate the immune response, the imbalance favoring Tconv cell activation and inflammation further promote immunological aging (33). Aging is known to result in a compromised ability of myeloid precursors to proliferate into T cells and an impaired response to cytokines. These factors might contribute to the significant increase in MDSC associated with the aging process (34) (Table 1).

**Table 1:** Summarization of evidences of immune tolerance mechanisms of children to SARS-CoV-2.

**Mechanism**	**Evidence (Reference)**
More robust innate immune response	Higher serum IFN-γ and IL-17A concentrations in pediatric patients protecting the children from progressive respiratory disease (5)
	Age-linked alteration in key pattern recognition receptor signaling pathways and cytokine responses including decreased antiviral interferon responses (6)
	Age related dysregulation of innate immune system including impaired cell migration and PRR signaling required for response to pathogen or vaccine (7)
	Lower concentration of IgG antibodies specific for the SARS-CoV-2 nucleocapsid (N) protein in children (8)
More cross-reactive T and B cell immunity	Higher proportion and number of T and B cells, and naïve T cells in children (11)
	Reduced lymphocyte counts in adults infected with SARS-CoV-2 (12)
	Frequent and recent infections by related coronavirus strains or recent vaccination might lead to more cross-reactive T and B cell immunity (13, 14)
Immunotolerance mechanisms	Lower concentrations of IL-6 and TNF-α in children (5)
	Lower frequencies of spike-specific CD4 T cells, neutralizing antibody titers, and antibody-dependent cellular phagocytosis leading to attenuated adaptive immune response in children to SARS-CoV-2 (5, 8)
	Rapidly changing environmental and microbial exposures during postnatal period leading to immune development and tightly regulated inflammatory reactions (22)
	Higher adaptive Treg cells as well as pronounced pattern-recognition receptors (PRR)-mediated IL-10 production by neonatal antigen presenting cells (24)
	ILCreg helps selective tolerance by producing IL-10 and TGF-b1 (25)
	CD71+ cells in neonates express multiple factors, including arginase-2, which are essential for immunosuppression in neonates (26)
	CD71+ cells promote the development and function of Tregs, which are more numerous in infants and children (27)
	A higher frequency of myeloid-derived suppressor cells in neonates (28)

Therefore, based on the available evidence we suggest an abundance of the immunosuppressive cells early in life may reduce COVID-19 severity. Consistent with this hypothesis, SARS-CoV-2 Tregs are reduced in hospitalized COVID-19 patients (35) and TGF-β+CD28- naïve CD8+ T cells are higher in patients with mild than severe COVID-19 (36). Interestingly, two patients with COVID-19 and acute respiratory distress syndrome were successfully treated with Tregs (37). However, there are currently no analyses of Tregs or other immunosuppressive cells in children with COVID-19, and this hypothesis needs to be explored further.

## Discussion

Evidence suggests that infants and children are susceptible to COVID-19 but show no or mild symptoms. Understanding how children circumvent severe COVID-19 would provide valuable information for understanding disease susceptibility and severity, as well as for developing treatment strategies for COVID-19. Although limited, most studies on children with COVID-19 focus on viral clearance mechanisms. We hypothesize that active immunotolerance contributes to milder disease in children with COVID-19, and more focus should be placed on understanding the immunotolerance mechanisms in children infected with SARS-CoV-2.

## Author Contributions

The conceptualization and writing of this article were performed by HE.

## Conflict of Interest

The author declares that the research was conducted in the absence of any commercial or financial relationships that could be construed as a potential conflict of interest.

## Publisher's Note

All claims expressed in this article are solely those of the authors and do not necessarily represent those of their affiliated organizations, or those of the publisher, the editors and the reviewers. Any product that may be evaluated in this article, or claim that may be made by its manufacturer, is not guaranteed or endorsed by the publisher.
